# The complete chloroplast genome sequence of *Leucanthemella linearis* (Matsum. ex Matsum.) Tzvelev (Asteraceae), endangered plant of Korea

**DOI:** 10.1080/23802359.2020.1821819

**Published:** 2020-09-17

**Authors:** Hyoung Tae Kim, Jung Sung Kim

**Affiliations:** a Institute of Agriculture Science and Technology, Chungbuk National University, Cheongju, Republic of Korea; b Department of Forest Science, Chungbuk National University, Cheongju,Republic of Korea

**Keywords:** *Leucanthemella linearis*, chloroplast genome, indels, regional specific marker

## Abstract

The complete chloroplast (cp) genome sequence of *LHeucanthemella linearis* was newly analyzed in this study. It was 151,395 bp in length and was a typical circular structure composed of a large single-copy region (LSC) (83,080 bp) and a small single-copy (SSC) region (18,391 bp) which were separated by two inverted repeat regions (24,962 bp). It was totally 6 bp shorter than the Chinese *L. linearis* cp genome and was composed of 132 genes. There were four regional specific Indels between them in the LSC region as well as 20 bp insertion in the intergenic spacer region excluding poly-A/T sequence variation. And it was clear that both of *Luecanthemella linearis* were sister to *Chrysanthemum* – *Artemisia* groups and their phylogenetic relationship from this study.

*Luecanthemella linearis* (Matsum. Ex Matsum.) Tzvelev is a member of the family Asteraceae and was treated as *Chrysanthemum lineare* Matsum. or *Leucanthemum lineare* (Matsum.) Vorosch. depending on the taxonomic recognition in the traditional classification. It is distributed in East Asia Manshuria, China, Korea, and Japan (El-Twab and Kondo 2007) against to the European native species *L. serotina*. Although just these two species comprise the genus *Leucanthemella*, they were scattered in the phylogenetic tree of *Chrysanthemum* and its related taxa (Masuda et al. 2009) and it needs comprehensive study for resolving this relationship. *Luecanthemella linearis* has generally large head flower with white ligulates and grows up to over 1 m. It is restricted inland marsh area with a less individual and was nominated as one of endangered species in the red data books of Korea due to its peculiar habitat and small population size (Korea National Arboretum 2009).

We obtained the plant material of *Leucanthemella linearis* which was collected from Mt. Chilbo-san of Suwon-si. Gyeonggi-do, Korea (37° 15.250′ N, 126° 56.060′ E, alt.204 M) and the voucher (CBNU2018-0242) was deposited at the Herbarium of Chungbuk National University (CBNU). Using the dried leaves samples, complete chloroplast (cp) genome of *Leucanthemella linearis* (MN883842) was sequenced by HiSeq4000 of Illumina and compared the sequence difference with Chinese *L. linearis* genome (NC043835). Totally 108,070,890 paired-end reads (2 × 151bp) were obtained and 100,955,336 reads were used for the assembly to the reference sequence after trimming with the length range 50–151 bp. The assembled reads were *de novo* assembled using the Geneious assembler. Using the assembled contigs, we conducted to align and repeat the procedure up to make a single contig. Complete cp genome was annotated using Geneious version 10.2.6 (Kearse et al. 2012) with manual correction and tRNAScan-SE (Lowe and Eddy 1997) for *tRNA* gene. The average coverage of this cp genome was 217.1×. The phylogenetic tree was constructed with related *Chrysanthemum* and *Artemisia* members and related Asteraceae based on the concatenated 78 coding genes using RAxML (Stamatakis 2014) after model test by ModelFinder (Kalyaanamoorthy et al. 2017).

The complete cp genome of *Leucanthemella linearis* has a typical circular structure with 151,395 bp in length and comprised a large single-copy region (LSC, 83,080 bp), a small single-copy region (SSC, 18,391 bp), and two inverted repeat regions (IR, 24,962 bp). It was totally 6 bp shorter than the Chinese *L. linearis* genome. The GC contents were 37.3%. It was composed of 132 genes and they were identified 87 coding genes, eight *rRNA* genes, and 37 *tRNA* genes. From the comparison of both *L. linearis* cp genomes, we found four regional specific Indels between them in the LSC region excluding poly-A/T sequence variation. They were 42 and 15 bp insertion on the present Korean *L. linearis* cp genome in *trnK* intron and intergenic spacer region of *trnT-psbD*, respectively, as well as 15 and 9 bp deletion in the intergenic spacer region of *trnK-rps16* and *petN-psbM*. In the inverted repeat region, there was 20 bp insertion in the intergenic spacer region between *ndhB* and *trnC* and 1 bp deletion of poly-A in the SSC region of Korean *L. linearis* genome. These indels will be useful for developing the molecular markers to detect regional variation of Korean and Chinese species.

From the result of molecular phylogenetic analysis, both of *Luecanthemella linearis* were sister to *Chrysanthemum* – *Artemisia* groups and their phylogenetic relationship were highly supported (Figure 1). It will provide more information for understanding the diversity and relationship of the family Asteraceae.

**Figure 1.**
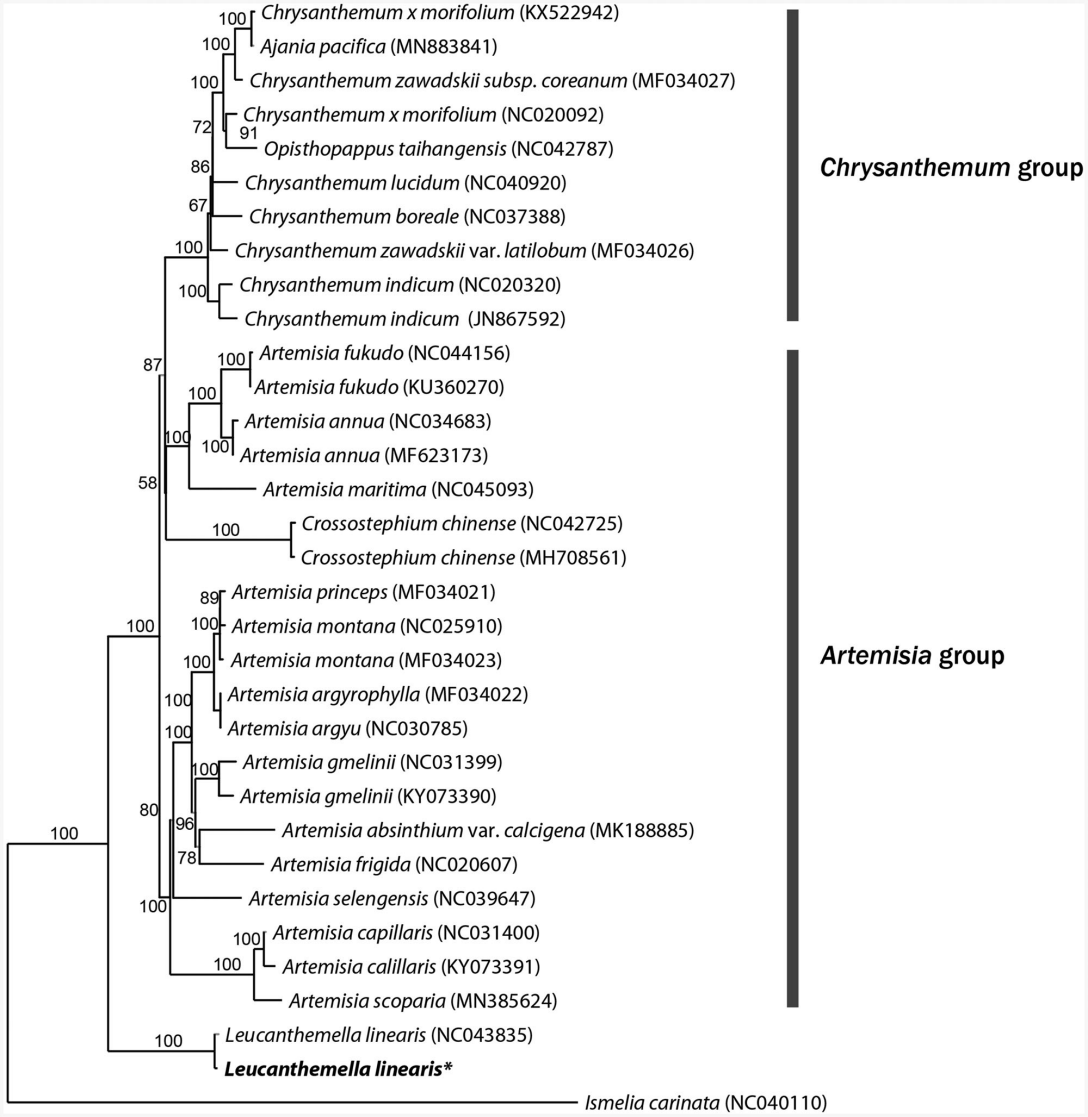
Phylogenetic tree of *Leucanthemella linearis* and related taxa using the complete chloroplast genome sequences.

## Data Availability

The data that support the findings of this study are openly available in NCBI database at https://www.ncbi.nlm.nih.gov/, reference number [MN883842] after this article is published.
